# Validation of a Non-invasive Inverse Problem-Solving Method for Stroke Volume

**DOI:** 10.3389/fphys.2021.798510

**Published:** 2022-01-26

**Authors:** Vasiliki Bikia, Carmel M. McEniery, Emma Marie Roussel, Georgios Rovas, Stamatia Pagoulatou, Ian B. Wilkinson, Nikolaos Stergiopulos

**Affiliations:** ^1^Laboratory of Hemodynamics and Cardiovascular Technology, Institute of Bioengineering, Swiss Federal Institute of Technology, Lausanne, Switzerland; ^2^Division of Experimental Medicine and Immunotherapeutics, University of Cambridge, Cambridge, United Kingdom

**Keywords:** vascular aging, cardiac output, mathematical modeling, data assimilation, non-invasive monitoring

## Abstract

Stroke volume (SV) is a major biomarker of cardiac function, reflecting ventricular-vascular coupling. Despite this, hemodynamic monitoring and management seldomly includes assessments of SV and remains predominantly guided by brachial cuff blood pressure (BP). Recently, we proposed a mathematical inverse-problem solving method for acquiring non-invasive estimates of mean aortic flow and SV using age, weight, height and measurements of brachial BP and carotid-femoral pulse wave velocity (cfPWV). This approach relies on the adjustment of a validated one-dimensional model of the systemic circulation and applies an optimization process for deriving a quasi-personalized profile of an individual’s arterial hemodynamics. Following the promising results of our initial validation, our first aim was to validate our method against measurements of SV derived from magnetic resonance imaging (MRI) in healthy individuals covering a wide range of ages (*n* = 144; age range 18–85 years). Our second aim was to investigate whether the performance of the inverse problem-solving method for estimating SV is superior to traditional statistical approaches using multilinear regression models. We showed that the inverse method yielded higher agreement between estimated and reference data (*r* = 0.83, *P* < 0.001) in comparison to the agreement achieved using a traditional regression model (*r* = 0.74, *P* < 0.001) across a wide range of age decades. Our findings further verify the utility of the inverse method in the clinical setting and highlight the importance of physics-based mathematical modeling in improving predictive tools for hemodynamic monitoring.

## Introduction

Over the last decade, hemodynamic monitoring has risen to the forefront of efficient and sustainable healthcare. Monitoring of biomarkers for vascular and cardiac function is a crucial factor in cardiovascular disease identification, treatment, and assessment of therapeutic response (Vincent et al., 2015). Stroke volume (SV) is a major biomarker of cardiovascular function, reflecting the interdependent performance of the heart and major blood vessels. Despite this, hemodynamic management of patients *via* SV remains limited and guided predominantly by simple brachial cuff blood pressure (BP) observations alone (Phillips et al., 2017). Such approaches compromise the utility and effectiveness of hemodynamically guided interventions (Thiel et al., 2009; Meng and Heerdt, 2016).

Clinically, the most reliable and accurate technique for cardiac output (CO) estimation is thermodilution, with SV derived by dividing CO by heart rate (HR). Although thermodilution is clinically feasible, it is highly invasive and associated with increased risk, and therefore is not suitable for routine investigation. To overcome these limitations, several less invasive methods for assessing CO and SV have been developed. Such methods include either minimally invasive techniques such as pulse contour analysis or oesophageal doppler, which are still relatively invasive and thus are excluded from the routine clinical examination, or non-invasive techniques such as inert gas rebreathing, doppler ultrasound or magnetic resonance imaging (MRI). The latter, while completely non-invasive and reasonably accurate, is expensive and requires costly equipment and expert technical staff (Porter et al., 2015). Moreover, none of these methods are practical for routine, continuous bedside monitoring of SV.

Recently, we proposed a mathematical inverse-problem solving method for acquiring non-invasive estimates of mean aortic flow using age, weight, height and measurements of brachial BP and cfPWV (Bikia et al., 2020). CfPWV can be routinely measured in clinical practice, has a satisfactory repeatability, and has been identified as an independent predictor of clinical outcomes (Laurent et al., 2006), making it a valuable adjunct to BP measurements in routine assessments of risk. Therefore, the required (input) measurements for our proposed method are simple and readily available from the clinic. Moreover, our approach relies on the adjustment of a validated one-dimensional (1-D) model of the systemic circulation (Reymond et al., 2009) and applies an optimization process for deriving a quasi-personalized profile of an individual’s arterial hemodynamics. As such, we believe it provides a more sophisticated method for SV estimation compared with traditional statistical modeling approaches. An initial clinical validation of the method was conducted in 20 healthy individuals against aortic flow data measured using ultrasound (Papaioannou et al., 2014), with the results indicating that the estimates of mean aortic flow were in good agreement with the reference ultrasound-derived flow values.

Following the promising results of our initial validation, we wished to validate our method using a more precise MRI-derived measure of SV in a larger group of individuals covering a wide age range. A second aim was to investigate whether the performance of our inverse problem-solving method is indeed superior to traditional statistical approaches using multilinear regression models.

## Materials and Methods

### Study Population

The dataset used for the current study was obtained from a previous investigation of MRI-derived regional aortic stiffness and diameter, as part of the Anglo-Cardiff Collaborative Trial (ACCT) (Hickson et al., 2010). Subjects were recruited from the Cambridge arm of ACCT and were free of clinical cardiovascular disease and medication. Approval was obtained from the local research ethics committee, and written informed consent was obtained from all participants.

### Protocol

All participants fasted for 4 h before any measurements were undertaken. Brachial cuff BP and cfPWV were measured after 10 min of supine rest. After a further 20 min of rest, participants entered the MRI scanner. Cine phase contrast magnetic resonance imaging (PC-MRI) sequences were then performed perpendicular to the aorta at the level of the ascending aorta, located 1 cm distal to the aortic valve. Image acquisition sequences and image analysis procedures have been described in detail elsewhere (Hickson et al., 2010) and have been summarized in the Supplementary Material. The MRI-derived SV values (SV_MRI_) were used as the reference data, against which the model-derived SV estimations (SV_inverse_) were compared. It should be noted that PC-MRI constitutes a very well validated technique and, most importantly, is considered as the non-invasive gold standard for SV derivation (Lotz et al., 2002).

### Arm Cuff Pressure and Pulse Wave Velocity

Brachial SBP (brSBP_oscillometric_) and DBP (brDBP_oscillometric_) were measured in duplicate in the non-dominant arm, according to the British Hypertension Society Guidelines using a validated oscillometric device (HEM-711A-E, Omron Corp., Matsusaka, Japan). CfPWV (cfPWV_SphygmoCor_) was measured using the SphygmoCor (AtCor Medical) device by sequentially recording electrocardiographic-gated carotid and femoral artery waveforms as previously described (Wilkinson et al., 1998).

### Inverse Problem-Solving Method to Estimate Stroke Volume

**FIGURE 1:**
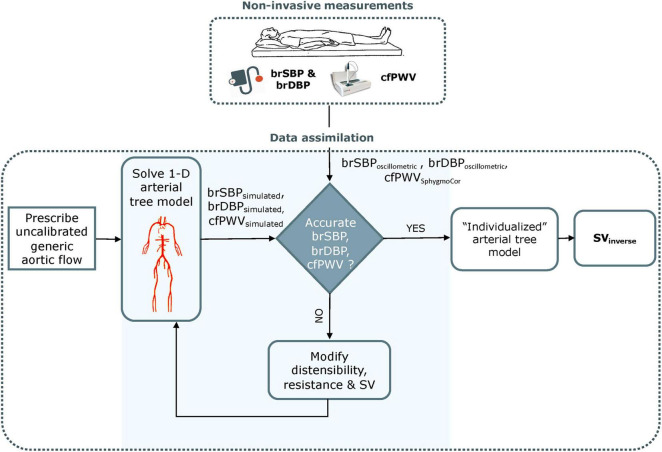
Schematic representation of the optimization process for estimating non-invasive stroke volume. brSBP, brachial systolic blood pressure; brDBP, brachial diastolic blood pressure; cfPWV, carotid-femoral pulse wave velocity; SV, stroke volume. Adapted from Bikia et al. (2020).

The inverse problem-solving method relies on an optimization algorithm in order to partially adjust a generic 1-D arterial tree model (Reymond et al., 2009; see Supplementary Material *1-D arterial tree model*) and to the specific participant under consideration (Figure 1). The rationale behind this approach was that adjusting some of the model parameters may be sufficient to approximate the measured data, namely brSBP_oscillometric_, brDBP_oscillometric_, and cfPWV_SphygmoCor_ (Watts and Bates, 1988).

The arterial tree model of this study is fully characterized by its geometry, the distensibility of all arterial segments and the peripheral impedances (described by terminal compliances and resistances). Additionally, aortic flow is needed as a proximal boundary condition. Identifiability analysis (Brun et al., 2001) demonstrated that, for any individual with a given set of brSBP, brDBP, cfPWV, HR, and SV values, there will be only one solution for the arterial tree model (Bikia et al., 2020). Therefore, if the generic arterial tree model modifies its parameters in order to approximate the measured brSBP, brDBP, and cfPWV, the model will approximate the hemodynamic profile of the participant under consideration and will yield a partially personalized model. This personalized model will allow for the derivation of SV.

#### Inverse Method

In applying our optimization algorithm, for an individual, the following information is required: gender, age, height, weight, brSBP, brDBP, HR, and cfPWV. In the first step, the method uses the demographic data (i.e., gender, age, height, and weight) for adjusting the geometry of the arterial tree model (see Supplementary Material *Anatomical adjustment of 1-D arterial tree model*).

The inverse method additionally accounts for the non-uniform aortic stiffening which occurs with aging (Kaess et al., 2012). For older individuals, stiffening is considered as non-uniform and more pronounced in the proximal aorta. This gradient in distensibility is adjusted by changing the relative regional distensibility of the proximal aorta through multiplication with an age-related proximal factor based on published literature (Kimoto et al., 2003). Subsequently, the heart cycle period (T_period_) is computed from the HR, whereas previously published data on the HR-related changes in systolic duration (T_systole_) (Weissler et al., 1968) are used to adapt the T_systole_ with respect to the measured HR. As a result, the only remaining flow-related parameter to be optimized for the aortic flow input is the aortic flow peak (Q_max_).

Following these model adaptations, the optimization algorithm is employed for adjusting the Q_max_, and the properties of the arterial tree, namely arterial compliance (C) and total peripheral resistance (R). An arbitrary parameter set of {C, R, Q_max_} is used in the first optimization iteration of the algorithm. Under all conditions, the 1-D model computes the simulated flows and pressure waves throughout the arterial tree, including the variables that correspond to the measured data (brSBP_oscillometric_, brDBP_oscillometric_, and cfPWV_SphygmoCor_) as well as the quantity of interest, namely the SV. The standard (non-optimized) model is expected to estimate inaccurate flows and pressures (and thus brSBP_simulated_ and brDBP_simulated_) due to the inaccurate input model parameters and the inaccurate input aortic flow for the specific individual under investigation. Similarly, the simulated cfPWV (cfPWV_simulated_) is not the same as the measured cfPWV_SphygmoCor_. To address this issue, the non-invasive, participant-specific measurements are integrated into the model using a gradient descent optimization algorithm. The reference C, R, and Q_max_ of the generic arterial tree are adjusted by multiplication with different scaling factors until the model-simulated brSBP_simulated_, brDBP_simulated_, and cfPWV_simulated_ (see Supplementary Material *Model-simulated pulse wave velocity*) are identical with the measured brSBP_oscillometric_, brDBP_oscillometric_, and cfPWV_SphygmoCor_. Once convergence is achieved, the simulated SV is considered as the final estimation for the specific participant. A more analytical description of the inverse problem-solving method can be found in the original publication (Bikia et al., 2020). The methodology described above was repeated for the entire study population (*n* = 144). The estimated SV_inverse_ were compared to the SV_MRI_. Accuracy was also assessed independently for the different age groups, i.e., 20–29, 30–39, 40–49, 50–59, 60–69, and ≥70 years.

Finally, we evaluated the errors resulting from the use of an approximated aortic flow waveform. We compared the T_systole_, Q_max_, as well as the time of Q_max_ (t_Qmax_) derived from the approximated flow waveform to the actual values extracted from the reference MRI aortic flow waveform. Consequently, we performed one-way analysis of variance (ANOVA) for the three estimated characteristics across the different age groups to investigate whether an age-dependent effect was observed.

### Multilinear Regression Analysis to Estimate Stroke Volume

In addition to the modeling analyses described above, we tested the performance of multilinear regression analysis using SV_MRI_ as the dependent variable. Overall, this approach allowed us to compare our inverse method with the more traditional multilinear regression method for estimating SV. For the multilinear regression method, the same parameters used as inputs to the inverse method were used as independent variables, namely age, gender, weight, height, HR, brSBP, brDBP, and cfPWV. We followed two different approaches for testing the performance of multilinear regression to: (i) a train/test split cross validation (CV) (1CV), and (ii) a 10-fold CV (10CV). For the 1CV approach, 100 out of the 144 participants were kept for defining the regression coefficients. Subsequently, the resulting regression equation was tested on the remaining 44 participants. This resulted in one multilinear regression model. The 10CV approach required that the group of 144 participants was randomly split into 10 equal subsets. One subset was allocated as the testing group to validate the regression equation, while the other nine subsets were used for defining the regression coefficients. This procedure was repeated 10 times so that all participants were used for testing. The performance metrics were derived by the average performance of all 10 models. The reason for adopting two CV approaches was to facilitate a more complete comparison between the two methods for estimating SV, i.e., inverse method and multilinear regression. We performed ordinary least squares (OLS) estimation of the regression coefficients using the statsmodels library (Seabold and Josef, 2010) for only 1CV setting. Hypothesis testing for each regression coefficient was realized using the *t*-statistic.

### Statistical Analysis

The statistical analysis was performed in Python (Python Software Foundation, Python Language Reference, version 3.6.8)^1^. All values are presented as means ± SD. The agreement, bias and precision between the model estimations (estimated data) and the reference data obtained from the MRI images were evaluated using the Pearson’s correlation coefficient (r), the mean absolute error (MAE), the normalized root mean square error (nRMSE) and Bland-Altman analyses (Bland and Altman, 1986). The computed nRMSE was based on the difference between the minimum and maximum values of the dependent variable (y) and was computed as RMSE/(y_max_–y_min_). Linear least-squares regression was performed for the estimated and reference data. The slope and the intercept of the regression line were reported. Two-sided *P*-values for hypothesis tests were calculated using Wald Tests with *t*-distribution of the test statistic. The null hypothesis was that the slope is zero. One-way ANOVA for unbalanced data (each group had different sample sizes) was performed on the estimations for the six age groups. A *P* < 0.05 was considered statistically significant.

## Results

Table 1 shows the subject characteristics of the study population (*n* = 144), including the MRI-derived SV reference data. The comparisons between the model-derived estimations for SV using (i) the inverse method and (ii) multilinear regression, and the reference SV data are presented below.

**TABLE 1:** Subject characteristics and hemodynamic parameters according to age group.

Parameter	All (*n* = 144)	20–29 years (*n* = 27)	30–39 years (*n* = 23)	40–49 years (*n* = 24)	50–59 years (*n* = 24)	60–69 years (*n* = 23)	≥70 years (*n* = 23)
Age (years)	49 ± 17	24 ± 3	34 ± 3	44 ± 2	57 ± 3	63 ± 2	74 ± 3
Gender (M/F)	62/82	11/16	12/11	9/15	10/14	9/14	11/12
Height (cm)	169 ± 10	172 ± 9	171 ± 9	169 ± 10	168 ± 9	169 ± 10	165 ± 10
Weight (kg)	70 ± 12	67 ± 11	73 ± 11	73 ± 15	68 ± 10	73 ± 13	68 ± 10
Brachial SBP (mmHg)	122 ± 16	112 ± 13	116 ± 9	120 ± 14	117 ± 12	128 ± 16	138 ± 16
Brachial DBP (mmHg)	71 ± 8	63 ± 4	68 ± 5	72 ± 9	71 ± 8	75 ± 6	75 ± 8
Brachial PP (mmHg)	51 ± 12	48 ± 12	48 ± 9	48 ± 8	46 ± 8	53 ± 13	63 ± 13
Mean arterial pressure (mmHg)	88 ± 10	79 ± 6	84 ± 6	88 ± 10	86 ± 8	93 ± 9	96 ± 10
Carotid-femoral PWV (m/s)	7 ± 2	6 ± 1	6 ± 1	7 ± 1	7 ± 1	8 ± 1	10 ± 2
Heart rate (bpm)	66 ± 12	68 ± 12	61 ± 9	66 ± 12	65 ± 11	66 ± 10	69 ± 14
Stroke volume (mL)	84 ± 21	92 ± 26	97 ± 17	90 ± 19	80 ± 16	79 ± 15	68 ± 11

### Estimation of Stroke Volume Using the Inverse Method

**FIGURE 2:**
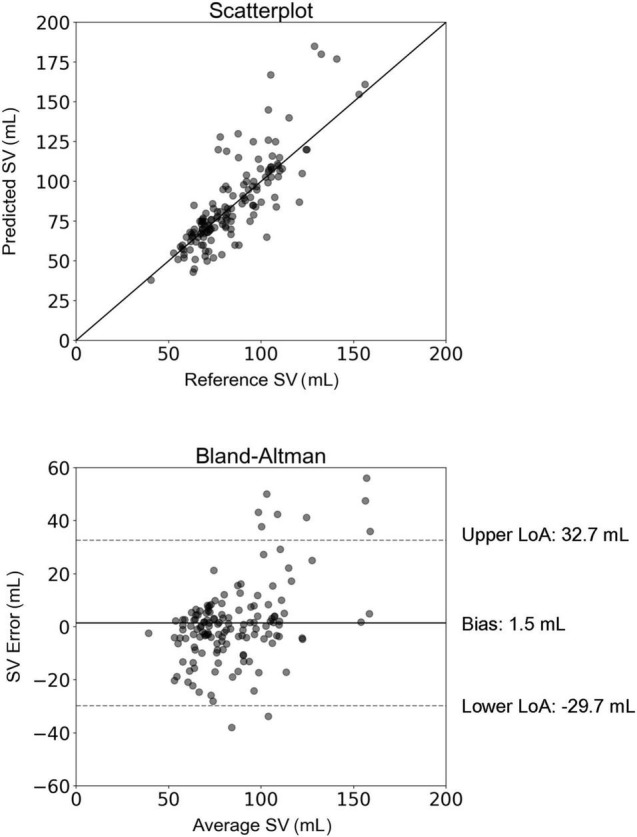
Scatterplot and Bland–Altman plot demonstrating the association between the estimated stroke volume (SV) (using the inverse method) and the reference SV (MRI). The solid line of the scatterplots represents equality. In Bland–Altman plots, limits of agreement (LoA) are defined by the two horizontal dashed lines.

**FIGURE 3:**
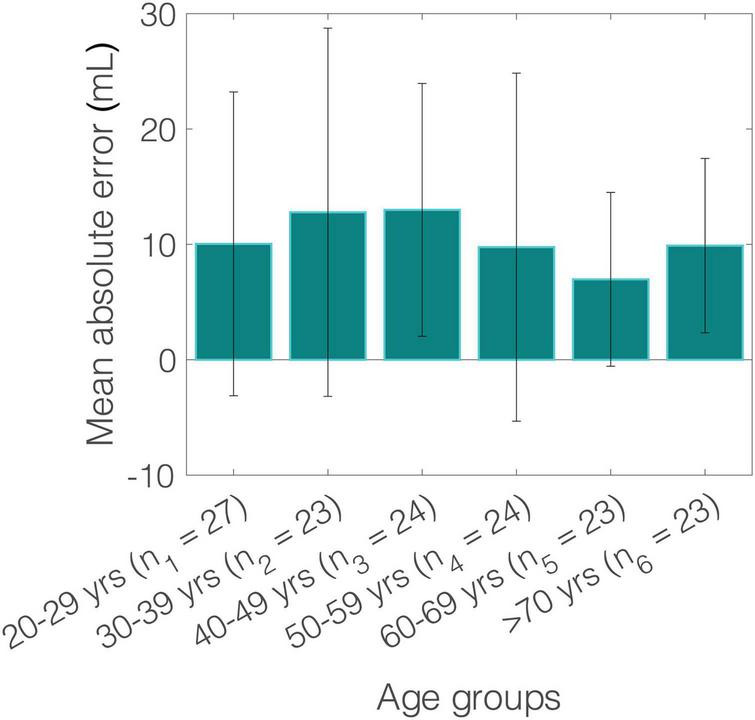
Variation of mean absolute error (MAE) of stroke volume (SV) across age groups.

The comparison between SV_inverse_ and SV_MRI_ is presented in Figure 2. The slope and intercept of the regression line were 1.1 (*P* < 0.001) and −8.8 mL, respectively. The nRMSE was 13.8%. Bland-Altman analysis yielded a low bias of 1.5 mL and limits of agreement (LoA) of (−29.7, 32.7) mL. The estimation error was outside of the LoA for only 7% of the study population. Variability of the mean difference between estimated and measured SV values was 15.9 mL. Although several overestimations were observed for high values of SV, the majority of the estimated data were tightly distributed around the line of equality (x = y). The MAE in SV estimation was computed for the different age groups of the study population (Figure 3). The overall variability of the MAE was ± 2.2 mL (*P* < 0.0001), while higher MAE values (>12 mL) were reported for participants aged between 30 and 49 years. Estimations of SV had the lowest errors for participants aged between 60 and 69 years. Overall, the MAE values differed significantly between age groups of the study population (*P* < 0.001).

### Approximated Aortic Flow Characteristics

Table 2 reports the measured (MRI) and estimated aortic flow characteristics for all participants and the different age groups. The estimated T_systole_ was slightly lower than the measured values for all age groups. The correlation between the estimated and measured data was *r* = 0.6 and the mean absolute percentage error was 10%. The estimation of Q_max_ was satisfactory with *r* = 0.7, and a small overestimation of the measured values. Finally, assuming a fixed aortic flow wave shape led to a less precise approximation of t_Qmax_ with a correlation coefficient of *r* = 0.41.

**TABLE 2:** Measured and estimated aortic flow characteristics for all participants and according to age group.

Mean ± SD															

	All (*n* = 144)		20–29 years (n = 27)		30–39 years (*n* = 23)		40–49 years (*n* = 24)		50–59 years (*n* = 24)		60–69 years (*n* = 23)		Decade >70 (*n* = 23)		*P*
								
	Real	Est	Real	Est	Real	Est	Real	Est	Real	Est	Real	Est	Real	Est	
T_systole_ (ms)	323 ± 58	296 ± 13	313 ± 46	294 ± 13	332 ± 57	302 ± 10	323 ± 40	296 ± 13	333 ± 97	297 ± 13	322 ± 33	297 ± 12	316 ± 55	292 ± 16	0.2
Q_max_ (ml/s)	400 ± 96	464 ± 129	448 ± 129	582 ± 129	441 ± 78	538 ± 120	417 ± 85	448 ± 98	368 ± 72	435 ± 99	386 ± 76	413 ± 93	330 ± 65	348 ± 72	<0.0001
t_Qmax_ (ms)	89 ± 23	122 ± 21	83 ± 19	119 ± 22	99 ± 23	131 ± 19	88 ± 19	121 ± 21	90 ± 26	123 ± 21	90 ± 23	121 ± 22	83 ± 27	117 ± 23	0.3

*SD, standard deviation; Est, estimation; T_systole_, systolic duration; Q_max_, peak of aortic flow; t_Qmax_, time at Q_max_. P-values were derived from one-way ANOVA for each estimated parameter (namely T_systole_, Q_max_, and t_Qmax_) across the six age groups.*

### Estimation of Stroke Volume Using Multilinear Regression Analysis

Hypothesis testing indicated that all of the specified coefficients, except for those corresponding to gender (*P* = 0.52) and brDBP (*P* = 0.28), were significantly different from zero. Therefore, the multilinear regression analysis was repeated, excluding gender and brDBP from the model.

The regression equation for the 1CV scheme was as follows:


$$\begin{array}{c} \text{SV}=-0.34\times \left(\mathrm{age}\right)+0.38\times \left(\mathrm{weight}\right)+40.14\times \left(\mathrm{height}\right)\\ \;+0.47\times \left(\mathrm{brSBP}\right)-0.45\times \left(\mathrm{HR}\right)-4.23\times \left(\mathrm{cfPWV}\right). \end{array}$$

**FIGURE 4:**
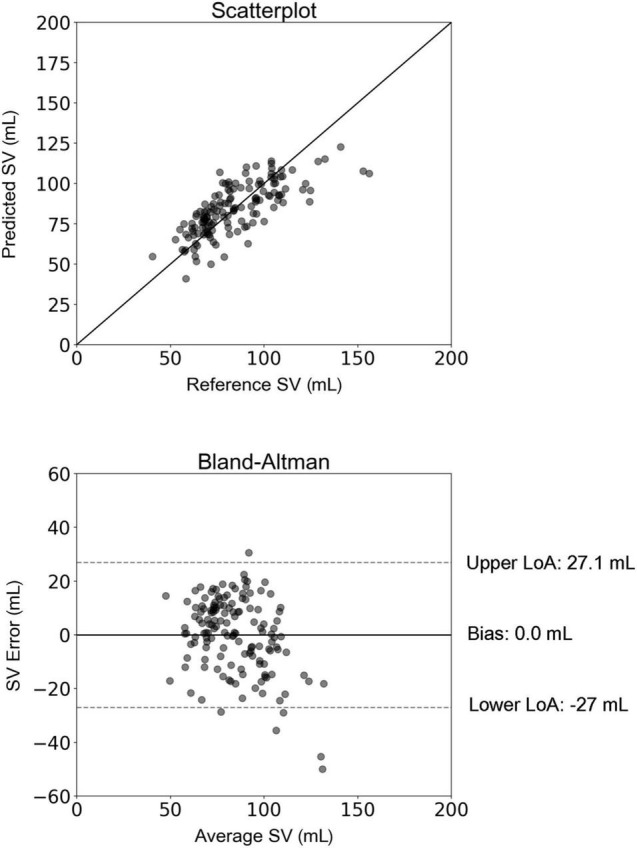
Scatterplot and Bland–Altman plot between the predicted stroke volume (SV) (using multilinear regression) and the reference (MRI) SV. The solid line of the scatterplots represents equality. In Bland–Altman plots, limits of agreement (LoA) are defined by the two horizontal dashed lines.

For the 10CV scheme, the comparison between the regression-estimated SV (SV_regression_) and the reference SV_MRI_ is presented in Figure 4. The slope and intercept of the regression line were 0.57 (*P* < 0.0001) and 36.32 mL, respectively. The LoA were equal to ± 27 mL and the bias was zero. Results of the new hypothesis testing for the OLS regression coefficients reported a *P*-value below 0.01 for all independent variables. Correlation and agreement between SV_regression_ values (using both testing schemes) and the reference SV_MRI_ values are presented in Table 3. Multilinear regression models yielded a lower correlation (*r* = 0.74) compared with the inverse method (*r* = 0.83), whereas the LoA were narrower in the case of multilinear regression analysis.

**TABLE 3:** Overall comparison among stroke volume (SV) estimates and reference MRI SV.

	mean ± SD (mL)	*r*	MAE (mL)	Bias (LoA) (mL)
Measured (*n* = 144)	84.4 ± 20.4	–	–	–
Measured (*n* = 44)*	82.6 ± 19	–	–	–
Inverse (*n* = 144)	86 ± 27.8	0.83	10.4	1.5 (−29.7, 32.7)
Inverse (*n* = 44)*	84.5 ± 26.1	0.85	10.1	1.9 (−25.4, 29.2)
MLR_10CV_ (*n* = 144)	84.5 ± 15.8	0.74	11	0.02 (−27, 27.1)
MLR_1CV_ (*n* = 44)*	84.6 ± 14.5	0.79	10.8	2 (−20.7, 24.8)

*MAE, mean absolute error; LoA, limits of agreement; MLR, multilinear regression; CV, cross validation.*

*1CV corresponds to train/test split equal to 100/44.*

*10CV corresponds to 10-fold CV.*

*Values correspond only to the test set (44 subjects).

## Discussion

In the present study, we validated a previously developed inverse problem-solving method for the estimation of a major hemodynamic parameter, the SV. The original method, based on non-invasive measurements of brachial BP and cfPWV (Bikia et al., 2020) underwent a preliminary validation in a small (*n* = 20) cohort of human subjects. Here, we have implemented and tested our method on a further 144 healthy individuals and compared the SV_inverse_ (estimated data derived from the inverse method) to SV_MRI_ (measured data derived from the non-invasive gold standard of MRI). Additionally, we have compared the performance of the inverse method against the predictive capacity of a traditional linear regression approach which uses the same set of inputs as those used in the inverse method. The two key findings of this study are that the inverse problem-solving method yields accurate estimates of SV across a wide range of ages and SV values, in a simple and cost-efficient manner in comparison to PC-MRI; and that a traditional statistical approach such as multilinear regression analysis is inferior to the more sophisticated inverse problem-solving technique, for a given set of clinical data.

The SV, together with BP, are fundamental and independent indicators of cardiovascular function and are essential for the understanding of cardiovascular physiology and pathology (Nichols et al., 2011). However, in clinical practice, BP and BP-derived surrogates of SV are often used either interchangeably with, or as replacements for, direct measurements of flow. This simplification potentially compromises our understanding of cardiovascular physiology and limits the clinical utility of hemodynamic analyses (Thiel et al., 2009; Asfar et al., 2014). While notable research efforts have been made for estimating SV using BP recordings (Jansen et al., 2001; Swamy and Mukkamala, 2008; Fazeli and Hahn, 2012; Ganter et al., 2016), none of these techniques accounts for the specific arterial tree properties unique to each individual.

Current doppler ultrasound technologies for SV in the clinical setting include echocardiography, transoesophageal doppler, and transcutaneous doppler. However, these techniques are associated with several limitations concerning applicability, cost and accuracy. For instance, transoesophageal doppler is largely limited to perioperative monitoring as the ultrasound transducer is inserted into the oesophagus and requires sedation. On the other hand, MRI allows for improved spatial resolution, larger imaging windows and higher tissue contrast than ultrasound-based techniques. Specifically, PC-MRI allows for accurate determination of the presence, magnitude, and direction of flow, as well as for the estimation of flow velocity, volume flow rate, and displaced volumes. In spite of these advantages, MRI remains inconvenient and expensive for routine examinations and requires long imaging times. As a result, monitoring SV effectively in a reliable, simple and cost-efficient way remains an unmet need.

Mathematical modeling of the human cardiovascular system offers valuable tools to investigate patient-specific aspects of arterial hemodynamics, which are difficult to assess in clinical practice. Data assimilation aims to address relevant challenges and can significantly promote patient-specific modeling (Wang et al., 2018). Rather than relying on simplified equations, we have followed a data assimilation approach, which is based on the adjustment of a generic 1-D arterial model using the non-invasive data of the peripheral cuff-based SBP, DBP, and cfPWV, which are easily obtained in a clinical setting. Successful tuning permits the creation of a personalized cardiovascular model which, consequently, provides access to key hemodynamic information including SV. The tuning is conducted *via* an optimization process which allows for the fusion between the computational model and the measured data. This study, along with the initial validation (Bikia et al., 2020), demonstrated that creating a partially personalized model can improve the prediction of SV.

Acquisition of cfPWV requires sequential recording of the carotid and femoral pressure pulse *via* applanation tonometry (Adji et al., 2011). CfPWV has a satisfactory reproducibility, while being an independent index of cardiovascular risk and/or mortality (Laurent et al., 2006). In our study, the role of cfPWV, as an index of arterial stiffness, was to facilitate the adjustment of the generic arterial tree model. Given that arterial distensibility, the inverse of arterial stiffness, constitutes a major parameter of the vasculature, combining the information provided by arterial stiffness and BP allowed us to determine aortic hemodynamics and thus SV.

The data from the ACCT allowed us to have an approximately equally split dataset for seven age decades, i.e., 20, 30, 40, 50, 60, and >70 years, which enabled an accurate comparison of the age-based results. Predictions of SV were precise across the different age groups, with a low variability of the MAE (± 2.2 mL). Lower errors were reported for the sixth decade of life. It was observed that the highest absolute errors corresponded to high values of SV, while predictions were more accurate for SV values below 130 mL. Overall, there was good agreement and high precision between the SV_inverse_ and the SV_MRI_ data across different age decades and SV values, which indicates a robust performance of the inverse method.

We also investigated the validity of the assumption of a fixed aortic flow shape by comparing the estimated values of T_systole_, Q_max_, and t_Qmax_ with their actual values. The inverse method relies on a previously published formula (Weissler et al., 1968) which provides a HR-related approximation of T_systole_. Overall, it was observed that the estimated T_systole_ values did not vary significantly between age groups, while the variability within the same age group was also rather small. Our results also indicated that the formula slightly underestimated the T_systole_ values. It is likely that this underestimation led to the overestimation of Q_max_. Given that the method yielded accurate estimates of SV, for achieving the same SV, an underestimated T_systole_ would naturally lead to an overestimated Q_max_. Finally, assuming a fixed shape for aortic flow wave resulted in deviations in the value of t_Qmax_ (mean absolute percentage error was equal to 47%). Despite the reported deviations in the timing features of the aortic flow wave, the estimated Q_max_ was in satisfactory agreement with the reference Q_max_. Given that our method aims to minimize the required inputs for estimating SV, the use of a fixed shape wave is a well-advised approximation. Nonetheless, future work will aim to personalize the aortic flow wave shape with respect to subject characteristics, such as age and gender.

Multilinear regression analysis was performed using two cross-validation approaches, namely 1CV and 10CV. Hypothesis testing was conducted, where the *P*-value for each independent variable tested the null hypothesis that the variable has no correlation with the dependent variable. Coefficients of gender and brDBP were not statistically significantly different to zero, indicating that there was insufficient evidence in our sample to conclude that a non-zero correlation exists. All other regression coefficients were reported to be statistically significantly different from zero.

We compared the inverse method with the conventional multilinear regression analysis. Comparison indicated a higher correlation for the former. The LoA were broader for the inverse method, which also reported a higher bias. This outcome was expected, if we consider that the regression equation was constructed using a subset of the study population. The MAE was lower for the inverse method. A notable advantage of the inverse method relies on its generalization ability. Statistical learning models (such as linear regression) are often prone to generalization issues. These models are dependent on the specific training data used for developing the regression equation, and while they are able to provide accurate estimates for a hold-out (not considered in the process of developing the regression model) test subset of the same dataset, they are not likely to perform adequately for other independent datasets (Shameer et al., 2018). This lack of accuracy might be attributed to differences in the measurement protocol (e.g., physician preferences, local care standards), medication selection or other clinical decisions which influence the model development (Shameer et al., 2018). Specifically, regression analysis requires prior knowledge of large sets of collected data in order to estimate the coefficients of the regression equation. On the other hand, the inverse method is able to offer improved performance without dependency on pre-defined, dataset-derived regression coefficients.

The limitations of the inverse method have been acknowledged in the original publication (Bikia et al., 2020). Moreover, the present study does not include validation of the method’s performance for continuous monitoring applications. According to a meta-analysis for a new method to equal or better the performance of thermodilution (invasive gold-standard technique), it should achieve a percentage error <30% (Critchley and Critchley, 1999). Although the percentage error addresses the accuracy requirement, it does not provide explicit assessment of the method’s ability for continuous monitoring, which is essential in critically ill or hemodynamically unstable patients. In this respect, the next step of this work is the validation of the method for continuous SV (or CO) monitoring. In addition, validation of our method is limited to a healthy population. In critical conditions (e.g., ICU), there might be extreme cases which may lead to abnormal hemodynamical interdependencies. We assessed the performance of the inverse method in a patient with diastolic dysfunction, which is a pivotal component of heart failure with preserved ejection fraction (HFpEF). Given the lack of relevant *in vivo* data in the literature, we tested a virtual subject which was generated using a computational model of diastolic dysfunction (Kadry et al., 2020). We evaluated an extreme case of diastolic dysfunction (the restricted phenotype) with brSBP = 127 mmHg, brDBP = 61 mmHg, HR = 75 bpm, cfPWV = 5.97 m/s, and SV = 83 mL. The inverse method yielded an estimate of 80 mL, suggesting that the proposed methodology might provide precise estimations for this pathological condition. Nonetheless, this cannot lead to a certain generalized conclusion and proper *in vivo* validation using diseased populations should be conducted. A simplified approximation approach was selected for modeling T_systole_. The rationale behind our approach relied on the effort to simplify the acquisition of the measurements required to estimate SV. The T_systole_ is not readily available in routine clinical practice (acquired from Ultrasound velocity recording for instance), and cannot be effectively modeled using the input measurements that we have at our disposal (namely SBP, DBP, and cfPWV). Therefore, this approach might come with a compromise in accuracy in the approximation of T_systole_. However, the sensitivity analysis that was performed in the original publication showed that T_systole_ is less sensitive in comparison to more prominent model parameters, such as the HR, C, R, and Q_max_) (Bikia et al., 2020). At large, this approach may be considered as a fair trade-off between simplicity and relative accuracy for SV estimation; as also indicated by the agreement between the estimated and the reference SV data. Another limitation pertains to the synchronization of the clinical measurements. In particular, contrary to the simulated data produced by the 1-D arterial tree model, which corresponds to completely simultaneous pressure and flow waves, the *in vivo* measurements were performed with a time difference. Nevertheless, the intervals between the measurements were rather short and therefore, we may deduce that there was not a high variation in the measured data. In addition, we used aortic flow data derived from PC-MRI as a reference method with which to compare our estimated SV values. Although PC-MRI is considered a well-validated method for aortic flow measurements, the invasive gold standard technique is thermodilution. Next validation steps will include testing our method against thermodilution-derived SV data. Finally, it should be clarified that this study compares the proposed inverse methodology against the most simplified version of a multivariate linear regression method. A more suitable regression model would account for non-linear relationships between the dependent and the independent variables. Nevertheless, such models, while being simplistic, may be commonly used in the clinical evaluation.

## Conclusion

We have demonstrated that SV can be estimated accurately from non-invasive, easily obtained clinical measurements of brachial cuff BP and cfPWV using an inverse problem-solving method. Values of SV estimated using our inverse method compared favorably with the reference SV data derived from PC-MRI. Importantly, agreement between predictions and reference values was higher with the inverse method than traditional linear regression. These results, along with the inherent generalization limitations of regression equations, highlight the importance of physics-based mathematical modeling in improving predictive tools for hemodynamic monitoring.

## Data Availability Statement

The data analyzed in this study is subject to the following licenses/restrictions: The data analyzed in this study involve human subjects and are not publicly available. Requests to access these datasets should be directed to CM, cmm41@medschl.cam.ac.uk and IW, ibw20@medschl.cam.ac.uk.

## Ethics Statement

The studies involving human participants were reviewed and approved by the Individuals selected at random from local general practice lists and open-access cardiovascular risk assessment clinics across East Anglia and Wales in the United Kingdom. Approval was obtained from the local research ethics committees. The patients/participants provided their written informed consent to participate in this study.

## Author Contributions

VB and NS conceived and designed the experimental protocol. CM and IW designed the clinical protocol and performed the measurements. VB developed the original algorithms, analyzed the data, ran the experiments, and drafted the manuscript. ER contributed to the analysis. All authors discussed the results and edited the manuscript.

## Conflict of Interest

The authors declare that the research was conducted in the absence of any commercial or financial relationships that could be construed as a potential conflict of interest.

## Publisher’s Note

All claims expressed in this article are solely those of the authors and do not necessarily represent those of their affiliated organizations, or those of the publisher, the editors and the reviewers. Any product that may be evaluated in this article, or claim that may be made by its manufacturer, is not guaranteed or endorsed by the publisher.

## Footnotes

1
http://www.python.org
