# Supplementary material for: Validation of a Non-invasive Inverse Problem-Solving Method for Stroke Volume
Source: Front Physiol. 2022 Jan 26;12:798510. doi: 10.3389/fphys.2021.798510 (PMC8826540; doi:10.3389/fphys.2021.798510)
Supplement: Supplementary file 1 [file Data_Sheet_1.docx]

Supplementary Material

This Supplementary Material provides additional information on the measurement protocol and methodological aspects of the non-invasive inverse problem-solving method to estimate stroke volume.

1. **Aortic flow measurements**

Images were acquired using a 1.5-T MRI system (Signa HDx, GE Healthcare, Waukesha, Wisconsin). An 8-channel abdominal/pelvic coil was placed over the subject lying supine and a cuff placed around the left arm for brachial BP measurement. Three plane localizer images were obtained to identify the ascending and descending aorta through to the bifurcation. A multi-slice, electrocardiographically triggered, black blood fast spin echo sequence was acquired in an oblique sagittal orientation to demonstrate the full length of the aorta. An electrocardiographically gated, segmented k-space, cine phase contrast sequence (PC-MRI) was used with the following parameters: 30° flip angle, 5-mm slice thickness, 280x280-mm field of view, 6.7 repetition time, 256x256 matrix, 2 excitations, and 150 cm/s through-plane velocity encoding, with 1 view per segment. The duration of each sequence was approximately 5 min, with a total acquisition time of approximately 30 min. One hundred temporal phases were retrospectively reconstructed with a true temporal resolution of 2.0 ± 6.7 ms due to the interleaved positive and negative velocity encoding.

PC-MRI images allowed for deriving the aortic flow waveforms. Data analysis was performed offline using CV Flow software (Medis, Leiden, the Netherlands). Aortic contours were automatically detected in each slice location to obtain aortic flow-time curves and aortic areas through the cardiac cycle. In addition, up-sampling to 1 kHz was performed by interpolation with custom software (version 2.6, Python Software Foundation, Wolfeboro Falls, New Hampshire). In turn, the aortic flow waves permitted the accurate computation of the SV values.

1. **Inverse problem-solving method for derivation of SV**
   1. **1-D arterial tree model**

In this study, we adopted a validated 1-D model of the systemic arterial tree that has been previously described by Reymond et al. [26]. The arterial tree includes the main arteries of the systemic circulation, including a network representation of the coronary circulation. In brief, the governing equations of the model are obtained by integration of the longitudinal momentum and continuity of the Navier-Stokes equations over the arterial cross section. Flow and pressure waves throughout the vasculature are obtained by solving the governing equations with proper boundary conditions using an implicit finite-difference scheme. The arterial segments of the model are considered as long tapered tubes, and their compliance is defined by a nonlinear function of pressure and location as proposed by Langewouters [1]. The arterial wall behaviour is considered to be nonlinear and viscoelastic according to Holenstein et al. [2]. Local arterial compliance (C) is calculated after approximating pulse wave velocity (PWV) as an inverse power function of arterial lumen diameter, following the physiological values reported in the literature. Resistance of the peripheral vasculature (R) and terminal compliances (C) are accounted for by coupling the distant vessels with three-element Windkessel models. At the proximal end, the arterial tree either receives a prescribed input aortic flow waveform or is coupled with a time-varying elastance model for the contractility of the left ventricle [3], [4]. In this study, we used a generic waveform with fixed shape as input to the arterial tree model. The aortic flow wave is characterized by three parameters, namely the heart cycle period (T_period_) the systolic duration (T_systole_), and the aortic flow peak (Q_max_). In order to decrease the computational cost of our method, we removed the brain circulation of the original 1-D model. Three-element Windkessel models were used as terminal boundary conditions at the left and right common carotid and vertebral arteries. Pressure and flow from the original configuration were used to derive the parameters of the three-element Windkessel models via fitting. The purpose of removing the cerebral circulation was to decrease the computational time of the simulation. The model has been thoroughly validated [5], [6] and is able to predict pressure and flow waves in good agreement with in vivo measurements. These waves can be used for pulse wave analysis techniques to derive several parameters of interest.

**2.2. Anatomical adjustment of 1-D arterial tree model**

The geometry of the 1-D arterial tree model is defined by the length and the diameter of each arterial segment of the tree. Arterial length is adjusted in accordance to height. The reference state of the arterial tree model corresponds to an individual with a height equal to 180 cm. Uniform adjustment of the arterial lengths is done via multiplication with a common scaling factor. Arterial diameter is uniformly adjusted based on previously published data that associate aortic diameter with age, gender, and BSA [7]. This completes the anatomical adjustment of the arterial tree model.

**2.3. Model-simulated pulse wave velocity**

CfPWV was derived using the foot-to-foot tangent method [8]. The method uses the intersection point of two tangents on the arterial pressure wave as a characteristic marker. The first tangent is defined as the line that passes tangentially through the initial systolic upstroke, i.e., the maximum of the first derivative. The second tangent line is the horizontal line passing through the minimum pressure point. By applying the method, the pulse transit time (PTT_simulated_) between the carotid artery and the femoral artery was estimated. Total arterial length was determined by summation of the lengths of the arterial segments within the transmission path, i.e., the relevant carotid-femoral path. Finally, simulated cfPWV (cfPWV_simulated_) was calculated by dividing the total length by the PTT_simulated_.

# Supplementary References

[1] G. J. Langewouters, *Visco-elasticity of the human aorta in vitro in relation to pressure and age*. Amsterdam: Free University of Amsterdam: Krips Repro, 1982. [Online]. Available: https://books.google.gr/books?id=qvSrHAAACAAJ

[2] R. Holenstein, P. Niederer, and M. Anliker, “A viscoelastic model for use in predicting arterial pulse waves,” *J Biomech Eng*, vol. 102, no. 4, pp. 318–325, Nov. 1980.

[3] K. Sagawa, H. Suga, A. A. Shoukas, and K. M. Bakalar, “End-systolic pressure/volume ratio: a new index of ventricular contractility,” *Am. J. Cardiol.*, vol. 40, no. 5, pp. 748–753, Nov. 1977.

[4] Suga Hiroyuki and Sagawa Kiichi, “Instantaneous pressure-volume relationships and their ratio in the excised, supported canine left ventricle,” *Circulation Research*, vol. 35, no. 1, pp. 117–126, Jul. 1974, doi: 10.1161/01.RES.35.1.117.

[5] P. Reymond, F. Merenda, F. Perren, D. Rüfenacht, and N. Stergiopulos, “Validation of a one-dimensional model of the systemic arterial tree,” *American Journal of Physiology - Heart and Circulatory Physiology*, vol. 297, no. 1, pp. H208–H222, Jul. 2009, doi: 10.1152/ajpheart.00037.2009.

[6] P. Reymond, Y. Bohraus, F. Perren, F. Lazeyras, and N. Stergiopulos, “Validation of a patient-specific one-dimensional model of the systemic arterial tree,” *Am. J. Physiol. Heart Circ. Physiol.*, vol. 301, no. 3, pp. H1173-1182, Sep. 2011, doi: 10.1152/ajpheart.00821.2010.

[7] A. Wolak *et al.*, “Aortic size assessment by noncontrast cardiac computed tomography: normal limits by age, gender, and body surface area,” *JACC Cardiovasc Imaging*, vol. 1, no. 2, pp. 200–209, Mar. 2008, doi: 10.1016/j.jcmg.2007.11.005.

[8] O. Vardoulis, T. G. Papaioannou, and N. Stergiopulos, “Validation of a novel and existing algorithms for the estimation of pulse transit time: advancing the accuracy in pulse wave velocity measurement,” *American Journal of Physiology-Heart and Circulatory Physiology*, vol. 304, no. 11, pp. H1558–H1567, Jun. 2013, doi: 10.1152/ajpheart.00963.2012.
